# Expert judgement of collaborative cloud classroom quality and its criteria using the many-facets rasch model

**DOI:** 10.1016/j.heliyon.2023.e20596

**Published:** 2023-10-05

**Authors:** Rahmi Ramadhani, Edi Syahputra, Elmanani Simamora, Soeharto Soeharto

**Affiliations:** a Doctor of Mathematics Education, State University of Medan, Medan, Indonesia; b Informatics Department, Universitas Potensi Utama, Medan, Indonesia; c Doctoral School of Education, University of Szeged, 30-34, Petőﬁ S. Sgt., Szeged, H-6722, Hungary

**Keywords:** Collaborative cloud classroom, Expert judgement, Multi-rater assessment, Many-facet rasch model

## Abstract

Collaborative Cloud Classroom (3CR) is an educational application that provides flexibility in the learning process. This specialized tool is designed to improve analytical and teambuilding skills, as well as enable students to document ideas, which are subsequently developed into bubble diagrams and text outlines. This study aims to assess and analyze the menu qualities possessed by the 3CR application using the Many-Facet Rasch Model (MFRM) analysis using 5 raters and 6 menus. A rating assessment was applied to 5 criteria, namely usability, functionality, visual communication, learning design, and security. In this case, a total of 135 from 150 data points were coded into FACETS software. The results showed that the six menus in the 3CR application were valid and reliable, as well as met the quality standards of learning tools. Security criteria were also the most difficult standards for raters to measure in all the application menus. The study also found that Rater A was more severe, and Rater E was more lenient in rating the 3CR menu. Rater B has overfitting and detected bias between rater gender and 3CR's menu (Discussion Forum and Learning Reflection menu) based on MFRM analysis.

## 1 Introduction

Education has become increasingly more digitized since the COVID-19 pandemic, altering learning patterns. This shows that a huge section of the global student population has reportedly been significantly impacted by the pandemic, concerning learning outcomes, social development and behavior, increasing educational gaps, and placing underprivileged pupils at risk of exclusion [1]. The pandemic has also transformed the learning process by completely using Information and communications technology (ICT) as the only new academic tool, to maintain ongoing educational activities. Furthermore, the development of educational digitalization is carried out in various lessons, such as learning mathematics. To observe the increased usage of digitalization in education for this mathematical knowledge, the utilization of various distance learning aids was necessary, such as the following (1) Learning Management System-Google Classroom [2], (2) Platform e-learning [3], (3) Kahoot [4,5], (4) Social media apps such as YouTube [6], WhatsApp [7,8], Telegram [9], and (5) Online meeting apps such as Zoom Meeting [10] and Google Meet [11].

The development of learning applications is also increasing with elevated usage since the outbreak of the Covid-19 pandemic. This development is continuously very important as a supporting component; to improve the quality of education according to the fourth goal of the Sustainable Development Goals (SDGs-4) is to ensure inclusive and equitable quality education and promote lifelong learning opportunities for all. Easy access to learning through the integration of technology in learning can increase lifelong learning opportunities for all students [12]. One of these developed applications is cloud classroom, which emphasizes the Internet of Things and Data Mining [13,14]. Data mining in the cloud classroom application assists students in dynamically selecting and organizing materials to be studied in the menu of the developed application so that students can learn independently. The cloud classroom application is also supported by the Internet of Things which provides information to students to see the learning path recommended by the system based on data on teaching materials. The recommended results are also adjusted to topics often discussed by students, so they are essential to study again. The collected data helps students evaluate their learning performance and prepare the next learning plan. Internet of Things and Data Mining used in the development of cloud classroom applications provide flexibility for students in determining the learning process according to their abilities and talents. Referring to the flexibility possessed by the Cloud classroom, it is suitable for use in emergency learning caused by the Covid-19 pandemic. It also supports the use of various blended learning models, such as FCM (flipped classroom model), which have been educationally applied after the Covid-19 outbreak.

The collaborative cloud classroom (3CR) application is one of the developments of the cloud classroom-based tool. We have developed the 3CR application in previous research, which can be accessed at the link http://3cr.6te.net/index.php. The development process of the 3CR application is done by utilizing the concept of cloud classroom, data mining, and the Internet of Things. We develop the 3CR application as a virtual class in implementing ethno-flipped classroom model-based learning. The development of the 3CR application in this research is still limited to a pilot study, so it needs to be evaluated further related to the validity of each application menu developed. The 3CR application developed is also still limited to the media used in implementing the ethno-flipped classroom (EFC) model. This limitation refers to the development of applications integrated with the stages of the ethno-flipped classroom model. The ethno-flipped classroom model is also a learning model developed by the researcher by integrating the flipped classroom model and the ethnomathematics approach. In this case, the development of the EFC model emphasizes the facilitation of flexible learning while still applying the traditional and cultural contexts of collaborative and structured group project activities [15]. It also categorizes learning into two stages, namely synchronous and asynchronous.

The 3CR application was developed because the EFC paradigm needs the assistance of the learning tools supporting both academic phases. The flexibility of this application is subsequently observed from the availability of several menus, including Content (C), Discussion Forum (DF), Project Result (PR), Test (T), Questionnaire (Q), and Learning Reflection (LR). Contrasting to the Cloud Classroom, the 3CR applications prioritize the availability of a collaboration in the Discussion Forum menu, which is flexibly designed by teachers for students' utilization. This is mainly utilized when carrying out the learning phase of group projects, according to the syntax of the ethno-flipped classroom model. Moreover, the development of the Collaborative Cloud Classroom application provides a new and meaningful learning experience for students. This is due to the superiority of the menu flexibility provided in building interactive, structured, tiered, and fair learning activities, to improve higher-order thinking and self-regulated skills.

The development of learning applications, especially educational integrative models, needs to meet the standard criteria, to ensure appropriateness and applicability in the learning process. According to Kustandi et al. [16], 3 criteria were described for developing learning applications, namely software engineering, learning design, and visual communication. The LD (learning design) aspect had several indicators, including learning design, material content, language, and communication. In the aspect of visual communication, seven indicators were observed, including communication, idea creativity, simplicity and attractiveness, audio, visual, moving media, and interactive layout (navigation). In testing the quality of developed learning applications, the use of other criteria also emphasizes the ISO 9126 standard, especially in the development of software applications. This standard applies five quality characteristics, namely functionality, reliability, usability, efficiency, and portability. Each of these characteristics subsequently produces sixteen sub-characteristics, such as suitability, accurateness, security, interoperability, maturity, fault-tolerance, recoverability, understandability, learnability, operability, attractiveness, time behaviour, adaptability, installability, coexistence, and replaceability [17].

Based on several characteristics or aspects used in the application quality assessment, we analyzed and determined five criteria related to the quality of the application developed. These set criteria were used in the six Collaborative Cloud Classroom (3CR) application menus, namely Usability (U), Functionality (F), Visual Communication (VC), Learning Design (LD), and Security (S). We have ensured that the criteria we have determined align with the supporting theories of technology-based learning application development described in the previous paragraph. The criteria and supporting theories must be appropriate so that the criteria used can measure the quality of the application developed. The assessment criteria are determined through the needs analysis and usage analysis of the 3CR application development. Furthermore, the application development quality was evaluated by raters using the five preset criteria. In this case, the assessment and analysis of the technological development were very important, due to their effects on the application's quality in the learning process. The specification and evaluation of software products (technology-based learning applications) were also key factors in ensuring that quality is achieved by defining the appropriate characteristics and considering the objectives of the developed outcome [17]. This was reinforced by a European survey, which generally analyzed e-learning quality, the use or implementation of related valuable instruments, and experience with various approaches. Based on the results, quality played a key role in the success of e-learning [18].

Many previous researchers have assessed product quality through expert evaluations. However, the analysis of expert judgment on product quality is still dominated using quantitative descriptive analysis by calculating the average assessment score given by the assessor and then adjusting to the assessment criteria. Quantitative descriptive analysis was used to assess the quality of open-ended-based worksheets [19]. The same analysis was also used by Rachmadtullah et al. [20], Yeni et al. [21], and Badu et al. [22]. Wesnawa et al. [23] used the Gregory formula to assess the quality of digital teaching materials. Supriyadi & Setiyawati [24] used Aiken's V formula to evaluate the training modules' quality. Other analyses were also used by Luque-Vara et al. [25] and Guillot-Valdés et al. [26] in assessing the quality of assessment instruments, namely using Fleiss' κ coefficient.

Various analyses can be used in assessing the quality of a product. However, the study used has yet to be able to investigate problems related to rater error in evaluating product quality. The role of raters in product quality assessment is crucial. Rater error in providing an evaluation will harm the product being assessed. The interaction between raters, the products being evaluated, the assessment items used, and the bias analysis have also not been able to be analyzed using traditional analysis approaches such as descriptive quantitative analysis. Based on this, an investigation is needed to investigate rater errors, rater interactions, and indications of bias that can occur in the assessment process. This research offers a Many-Facet Rasch Model (MFRM) analysis used to assess product quality, namely the 3CR application we developed.

Multi-faceted Rasch measurement refers to a class of measurement models suitable for simultaneously analyzing multiple variables that potentially impact assessment outcomes. MFRM models, also known as facets models, incorporate more variables, or facets, than the two variables included in classical testing situations [27]. Product quality assessment contains aspects of the product items being assessed, the assessment criteria items used, and other elements such as raters. Other factors likely to influence rater assessment can also be used in determining product quality, such as gender, educational background, length of work, and age. Relevant factors that influence the evaluation are usually called facets.

Another advantage of MFRM analysis is that it can measure rater severity (or leniency), assess rater consistency, correct examinee scores for differences in rater severity, examine rating scale functions, and detect potential interactions between different aspects [28]. The Many-Facet Rasch Model analysis is often used in the assessment of specific performance or skills, and this analysis is often used in language and management. Based on this, the use of MFRM analysis is still not used in assessing product quality, so we used MFRM analysis in conducting 3CR application quality assessment by using rater analysis, considering application menu, and assessment criteria as facets in MFRM analysis. As an additional analysis, we also used the distal facet, which is the gender rater, and acts as a dummy facet in detecting gender rater bias on the 3CR menu quality assessment. The use of MFRM analysis in this study will significantly impact product quality assessment research that considers the factors of rater error, rater bias, rater severity/softness in rating, and other analytical factors.

### 1.1 Research questions

This study aims to evaluate and analyze the menu qualities possessed by the 3CR application using the MFRM analysis, based on the standard criteria for developing learning applications. The three emphasized study questions are as follows.

1. How are the validity and reliability of 3CR's menus and criteria based on Rasch parameters?
2. What are the rater measurement results toward the 3CR's menu and the assessment criteria?
3. How are raters' severity and leniency in determining 3CR quality?
4. To what extent is evidence of gender bias in the 3CR's menu based on raters' evaluation?

## 2 Methods

### 2.1 Participants

A quantitative approach was used to assess and analyze the menu quality possessed by the 3CR application. The assessment was carried out through five raters, which contained the raters having qualifications in the fields of ET (Educational Technology), Computer Science, as well as Technology and Vocational Education. The code for each rater was A, B, C, D, and E, with the demographic data of raters being presented in Table 1.

**Table 1:** The demographic profile of raters in this study.

Demographic		Frequency	Percentage (%)
Gender	Male	3	60
	Female	2	40
Age	below 40 years old	1	20
	40–45 years old	2	40
	46–50 years old	2	40
Major	Computer Sciences	2	40
	Educational Technology	2	40
	Vocational Education Technology	1	20
Work Experience	Below 15 years	1	20
	15–20 years	2	40
	20–25 years	1	20
	26–30 years	1	20

### 2.2 Instruments

The instrument used in this study was an application quality assessment rubric consisting of assessment criteria using a four-rating scale (1 = strongly insufficient, 2 = insufficient, 3 = sufficient, and 4 = appropriate) (see Appendix A). The item criteria used to assess the menus in the 3CR application totaled 30 items (consisting of 5 criteria on 6 menus). The compiled criteria were also used to assess all the menus contained in the 3CR application. In this analysis, a total of 6 menus (Content, Discussion Forum, Project Result, Test, Questionnaire, and Learning Reflection), 5 criteria (Usability, Functionality, Visual Communication, Learning Design, and Security), and 5 raters were appropriately utilized. This indicated that a total of 150 data (6 menus x 5 criteria x 5 raters) were obtained from the analysis, without the occurrence of missing parameters.

### 2.3 Procedures

In the field of education, we administered a training session for evaluators to familiarize them with the tool and the scoring criteria used in the rubric, since quality assessment in the 3CR application depends on the perception of the evaluators. Rater training is essential before the raters conduct the actual assessment of the 3CR application menus. Rater training was conducted to overcome the problem of rater variability. Research has shown that well-trained and experienced raters exhibit systematic differences in their interpretation of rating criteria. When interrater reliability is sufficiently high, it indicates that raters have a consistent understanding of the construct and rating scale. As a result, they are more likely to provide accurate ratings that are consistent with the “high” quality level of the 3CR menus [27]. Previous research has shown that raters can differ not only in the strictness or leniency with which they judge quality, but also in how closely they follow scoring guidelines. There may also be differences in how they interpret the scoring criteria, their understanding and use of the rating scale categories, and the extent to which their ratings are consistent across various aspects such as scoring criteria, tasks, timing of the assessment, and other relevant factors [29].

An assessment session, practical use of the 3CR apps to be assessed, and an introduction to the assessment rubric were all part of the rater training. The raters were presented with the criteria for evaluating the application's quality. During the early stages of the training, they explored several cloud-based classroom applications, including Google Classroom, CCR, and similar alternatives. In this session, the researcher also shared perceptions with the raters regarding the types of cloud-based classroom applications to be studied and the application quality assessment criteria used. The perception equation provides information on what points the raters need to consider when assessing later. Furthermore, the raters were given three weeks to study and compare several other cloud-based classroom applications.

In the subsequent phase, the raters received instruction on utilizing scales within the assessment tool, which encompassed various subscales. This was followed by a hands-on assessment session in which one of the raters evaluated one of the alternative cloud-based classroom applications they had previously studied. Raters were asked to assess the quality of the 3CR application using the assessment tool after the training session. The assessors were given four weeks to analyze and assess the quality of the 3CR application. The assessors were given access to the 3CR application and were asked to assess the quality of each menu presented in the 3CR application. The researcher asked the assessor to fill in information related to the gender of the assessor in the assessment instrument. At the end of the program, the 3CR application was successfully assessed by five assessors. This study collected 5 raters x 6 3CR menus assessed x 5 assessment criteria used = 150 data points without missing data. We utilized the FACETS software to conduct MFRM analysis on this dataset (please refer to Appendix B for details).

### 2.4 Data analysis

The obtained data were analyzed using the Many-Facets Rasch Model (MFRM) analysis, which is a development of the RMM (Rasch Model Measurement) carried out for multi-assessment tests [30]. This analysis was developed by Linacre (1989), to adjust the introduced rating variabilities through the use of multiple raters [31]. Using the MFRM analysis, each rater is appropriately modelled according to the usability of a rating scale, where the model does not expect identical responses [32]. This analysis subsequently allows raters to provide different assessments. It has also been used in many studies to address rater-related variability and inconsistency within various fields [33,34].

All data, which consisted of quality assessment rubric ratings, were analyzed via the latest FACETS application version 3.84.0 [35]. Our data analysis began with the calculation of raw scores derived from the rater ratings, which were then imported into Microsoft Excel. We then developed a special coding system (see Appendix A) to facilitate multi-rater analysis using FACETS. The initial standard version of the mathematical model was adjusted to account for gender interactions. The subsequent equation represents the revised model for MFRM analysis:

(1) $$\ln\left[\frac{P_{nljgmk}}{P_{nljgmk-1}}\right]=B_{n}-D_{l}-G_{j}-C_{j}-E_{k}$$

where:

$P_{\text{nljgmk}}$ = probability of menu n receiving a rating of k from rater j with gender g majoring in m on task l.

$P_{\text{nljgmk}-1}$ = probability of menu n receiving a rating of k−1 from rater j with gender g majoring in m on task l.

$B_{n}$ = ability of menu n

$D_{l}$ = difficulty level of scoring criterion l

$G_{j}$ = the gender of rater j

$C_{j}$ = the level of seriousness exhibited by rater j

$E_{k}$ = the level of difficulty for the threshold between categories k−1 and k on the scale, specific to the evaluation criterion labeled as “l” [27,32]

The MFRM model used in this study is a model that has three facets (rater, criteria, and 3CR's menu). The MFRM model presented in Equation (1) is called the three-facet rating scale model. The measurement units are shown in logits, ranging from minus infinity (−∞) to plus infinity (+∞). Both 3CR's menu quality and item difficulty are expressed on this scale. Generally, the logit is the measurement unit of the scale for any parameter specified in a Rasch model. All facets will be represented in the log-odd unit (logit), an interval scale.

The MFRM analysis measures the interactions between the aspects capable of signalling unexpected responses or bias in the assessment process. It also detects other rater impacts through specific fit statistics, such as range limitation, halo effect, and internal consistency [36]. This analysis facilitates the observation and calibration of differences in rater severity levels, subsequently enabling interpretative considerations. Based on the severity (*C*) of the rater (*j*), the difficulty level of each aspect was calibrated. This severity was considered an estimate of the study subject (*n*) probability, which answers item (*i*) for the threshold parameter (*k*) on the rater (*j*) [37].

The threshold parameter (k), based on equation (1), indicates how the raters will handle the rating data. The parameter specifies that an MFRM should be used across all facet elements. The threshold parameters are calibrated jointly and using the same rating scale across raters, rater's gender, criteria, and menu in the 3CR application. Furthermore, as shown in equation (1), there is the term rater severity (*Cj*) [27]. Rater severity is present when raters provide ratings that are consistently too harsh compared to other raters or established benchmark ratings (i.e., consensus ratings provided by a group of raters for specified levels of menu ratings).

Precisely, in Equation (1), the parameter Cj models the severity of rater j; the greater the value of this parameter, the lower the rating is predicted to be. Put differently, the model in MFRM implies that severe or harsh raters tend to award lower scores to 3CR's menus, indicating a lower 3CR's menus rating. Conversely, lenient raters tend to award higher scores. The assessment's degree of severity and leniency will be determined for each facet [27,32].

The second step of data analysis is assessing the validity criteria. Fit statistics were evaluated based on the mean-square infit statistic (Infit MNSQ), mean-square outfit statistic (Outfit MNSQ), standardized infit statistic (Infit ZSTD), and standardized outfit statistic (Outfit ZSTD). Infit and outfit mean-square statistics (Infit and Outfit MNSQ) have an expected values range from 0.5to1.5. The facet element's infit and outfit ZSTD values are acceptable if between −2and+2. Significantly negative ZSTD values (ZSTD≤−2.0) indicated data overfit, significantly positive ZSTD values (ZSTD≥−2.0) indicated data misfit. According to Vahid et al. [38] standardized fit statistic (ZSTD) test that the data fit the model “perfectly”, whereas mean-square fit statistic (MNSQ) indicated whether the data fit the model “usefully”. Note that the ZSTD value is highly influenced by the sample size. Nevertheless, in order to attain a dependable ZSTD value, a suitable sample size should be less than 250. ZSTD values exceeding a sample size of 250 can be disregarded [[39], [40], [41]]. Therefore, the ZSTD value in this research can be ignored, as the number of data samples analyzed is less than 250 (the data analyzed in this research is 150 data).

In this research, we also employed the chi-square test within FACETS to assess statistical significance. The Chi-square test is employed in the context of the 3CR to discern between different levels of menu quality, criteria difficulty, and rater severity. A large p-value suggests that the level is characterized by a similar rather than a distinct one (e.g., p<0.05or0.01).

The third step of data analysis is to evaluate the rating scale used in the quality assessment of 3CR's menu. The performance of the rating scale was assessed using Rasch-Andrich thresholds require that the mean scores of the rating groups consistently improve from lower to higher criteria [27,40]. There are two types of facets in MFRM analysis, namely, the proximal facet and the distal facet. The excess contained in the 3CR's menu represents the construct to be measured, called the proximal facet. The rater's background, such as gender, is called the distal facet. The final analysis was performed on the interactions that occur between facets. Facets can interact with each other, such as the interaction between distal facets (gender) and proximal facets. Examples of interactions between facets that occur in this study are when the rater's gender interacts with the 3CR menu, or when the rater's gender interacts with the rater's interpretation of the evaluation criteria [27,35].

The outcomes arising from the interplay among facets yield an interaction bias, which can be assessed using model bias facets. Bias analysis examines how interactions between demographic factors, such as gender, affect the validity of assessments. The Differential Item Functioning (DIF) graph illustrates the interaction associated with rater bias. The analysis of DIF can be confirmed by the DIF contrast and a statistically significant probability (p < .01) [42].

## 3 Results

### 3.1 Fit validity and reliability (RQ1)

Analyzed in this study were the 30 criteria items using fit analysis, which is detailed in Table 2. The criteria items presented in Table 2 below are used to assess the six menus of the 3CR application. The criteria used in assessing the six menus of the 3CR application amounted to five criteria, so the required criteria items were 30 items (6 menus x 5 criteria = 30 criteria items).

**Table 2:** Measurement result for the criteria item.

Criteria Item	Total Score	Measure (Logits)	SE	In. MNSQ	In. ZSTD	Out. MNSQ	Out. ZSTD	PTMAC
1	17	−0.25	0.81	0.38	−1.19	0.39	−1.17	0.79
2	19	−1.95	1.12	0.66	−0.24	0.46	−0.28	0.63
3	18	−0.97	0.89	1.20	0.53	1.10	0.37	−0.04
4	15	0.94	0.74	0.20	−1.70	0.21	−1.68	0.00
5	15	0.94	0.74	1.77	1.18	1.77	1.19	0.43
6	20	−3.33	1.89	1.00	0.00	1.00	0.00	0.00
7	20	−3.33	1.89	1.00	0.00	1.00	0.00	0.00
8	19	−1.95	1.12	0.96	0.21	0.77	0.12	0.28
9	17	−0.25	0.81	0.48	−0.89	0.48	−0.90	0.66
10	15	0.94	0.74	1.77	1.18	1.77	1.19	0.43
11	19	−1.95	1.12	0.66	−0.24	0.46	−0.28	0.63
12	19	−1.95	1.12	1.21	0.51	1.32	0.63	−0.11
13	19	−1.95	1.12	1.21	0.51	1.32	0.63	−0.11
14	16	0.37	0.77	0.68	−0.35	0.74	−0.26	−0.06
15	15	0.94	0.74	1.05	0.30	1.06	0.31	0.25
16	17	−0.25	0.81	1.52	0.95	1.62	1.08	−0.75
17	15	0.94	0.74	0.20	−1.70	0.21	−1.68	0.00
18	16	0.37	0.77	0.68	−0.35	0.74	−0.26	−0.06
19	13	1.95	0.68	0.71	−0.33	0.68	−0.39	0.15
20	11	2.85	0.67	0.83	−0.13	0.80	−0.20	0.59
21	17	−0.25	0.81	1.55	0.99	1.44	0.85	0.60
22	19	−1.95	1.12	1.12	0.40	1.06	0.41	0.06
23	20	−3.33	1.89	1.00	0.00	1.00	0.00	0.00
24	16	0.37	0.77	0.16	−1.94	0.18	−1.88	0.87
25	14	1.46	0.71	0.72	−0.29	0.75	−0.22	0.83
26	16	0.37	0.77	1.25	0.57	1.25	0.57	0.55
27	18	−0.97	0.89	0.68	−0.44	0.63	−0.43	0.56
28	17	−0.25	0.81	0.64	−0.51	0.61	−0.57	0.48
29	15	0.94	0.74	1.63	1.03	1.60	1.01	0.57
30	14	1.46	0.71	2.49	1.90	2.36	1.78	0.65

SE = Standard Error, PTMAC = Point measure correlation.

Based on the criteria outlined in Table 2, item 20 appears to be the most difficult. In this context, the raters consistently assign a lower score to the 3CR menu, with a measure (logits) of 2.85 and a standard error (SE) of 0.67. Conversely, criteria 6 and 7 are the least demanding, with raters generally giving the 3CR menu a high score, with a measure (logits) of −3.33 and a standard error (SE) of 1.89. Table 2 also shows that the total score given by all raters on each item of 3CR's menu assessment criteria is close to the maximum total score. The total score shown in Table 2 is the total assessment score given by all raters on each item of 3CR's menu assessment criteria. Other information shown in Table 2 is the Measure (logits) column. The Measure column is a column that provides information on the logit value obtained on each criteria item used in assessing the quality of the 3CR menu. The estimates of ability and difficulty that result from a Rasch analysis are referred to as logits [43]. They are defined mathematically as the natural log of an odd ratio. The logit function shows the measurement process with equal intervals. The logarithm function changes the value from 0.01, namely log (0.01) to −2.0, log (0,1) value to −1.0, and log (1) to 0. Based on this, the logit value can be used as a reference for measurement in forming a scale obtained from observation or assessment [39].

The analysis continued to look at fit validity which can be obtained from Table 2 above. The average values of the infit and outfit MNSQ across the facets of the criteria items ranges from 0.16 logits to 2.49 logits. This suggests that we have not achieved the desired fit validity. The unfit items were observed on the Infit MNSQ (criteria item number 1, 4, 5, 9, 10, 16, 17, 21, 24, 29, and 30) and the Outfit MNSQ (criteria item number 1, 2, 4, 9, 11, 17, and 24). The appropriate range is between 0.5 and 1.5, with excellent fit criteria values near 1.00 logits [44,45]. The average of the infit and outfit ZSTD across all facets also meets the fit validity criteria, with the threshold ranging from −1.88 logits to 1.90 logits. The acceptable criteria range should be from −2 logits to 2 logits [46,47].

In terms of infit and outfit MNSQ scores, nine criterion items had scores within the acceptable range. Specifically, these criterion items are 1, 2, 4, 9, 11, 17, 19, 24 and 29. We retained these items for analysis due to their positive point-measure correlation (PTMAC). This decision is in line with the approach recommended by previous studies, as it helps to evaluate menu measures at different levels [40,48]. Point measure correlation is the Pearson correlation between observed scores, and combined measures provide additional information on the correspondence between observation and model expectations [27].

Criteria item 16 has infit MNSQ values closed to the threshold (0.5–1.5) with 1.52. However, we considered doing item retention because the criteria, item 16, is close to the threshold. In other Rasch research contexts, some researchers still include the item with infit and outfit MNSQ below 1.6. We did a different thing for item criteria 5 (item criteria: Content Security), 10 (item criteria: Discussion Forum Security), and 30 (item criteria: Learning Reflection Security), where these three items were excluded from the data analysis. This decision referred to the Infit MNSQ values on the three criteria items that were too far beyond the range (0.5–1.5). Based on this analysis, we use 27 criteria items for further analysis.

In the subsequent MFRM analysis, all of the items criteria subsequently possessed good unidimensionality, concerning the variance of at least 40% in the Rasch Model [49,50]. In this process, the variance obtained was 48.10%, indicating that all the utilized criteria items within the assessment rubric measured one dimension as 3CR application quality.

Table 3 shows the Rasch parameters derived from the MFRM analysis across the three primary facets. We will use the distal facets, specifically the gender of the rater, to illustrate the bias interaction.

**Table 3:** The statistical overview of the MFRM analysis.

Statistic	Menus	Criteria	Raters
Measure			
Mean	.00	0.00	−2.85
SD	1.07	1.42	0.67
FAM			
Mean	3.38	3.38	3.38
SD	0.28	0.38	0.18
SE	0.45	0.41	0.41
Outfit MNSQ			
Mean	0.91	0.95	0.92
SD	0.25	0.22	0.33
Infit MNSQ			
Mean	0.97	1.01	0.99
SD	0.25	0.25	0.29
Outfit ZSTD			
Mean	−0.10	−0.10	−0.10
SD	0.70	0.60	1.00
Infit ZSTD			
Mean	−0.10	0.00	0.00
SD	0.90	0.90	1.00
Strata	2.10	4.79	2.09
Separation ratio	2.96	3.34	1.31
RMSE	0.46	0.41	0.41
Separation (strata) Index (*H*)	3.43	4.95	2.51
Reliability	0.82	0.92	0.63
Separation Reliability (*R*)	0.84	0.92	0.73
Chi-square/df	33.5/5*	56,6/4*	10.9/4*
Inter-rater reliability			
Observed Exact Agreements			55.6%
Expected %			55.6%

*p < .01, SD = Standard Deviation, SE = Standard error, FAM = Fair average measure.

The acceptable range for the reliability criteria is between 0.68 and 1.00 [40,46]. Fisher [46] indicated that reliability is considered acceptable when values exceed 0.67. The facets show reliable scores within an acceptable range, with menus scoring 0.82 (in the “good” category) and criteria scoring 0.92 (also in the “good” category) [31,51]. Chi-square test results for all facets showed statistically significant p-values. Thus, each component of the model is measured at a separate (unequal) level.

Furthermore, the separation (strata) index (H) of the criteria items, menus, and raters were 3.43, 4.95, and 2.51, respectively. The menu segregation, or the number of menu strata, the index provided the number of different measurable levels of 3CR's menu quality. According to Table 3, the value of the menu separation index was 3.43, indicating that there were approximately three statistically distinct classes of menu quality among the six menus in the 3CR application included in the analysis. This result does not correspond to the number of measurement scales used, even though the menu quality measurement scale has four ratings. According to this analysis, more than one rating scale is needed to measure menu quality.

Another result is seen in the value of the rater separation index. The value of 2.51 suggests that among the five raters included in the analysis, there were only three statistically distinct classes of rater severity - which is more than expected when adopting the standard view with its fundamental objective of employing raters drawn from a group that is as homogeneous as possible. Concerning the criterion facet, the separation index attained a value greater than four (4.95) and, thus, more significant than the number of criteria included in the analysis. This result indicates that the spread of the criterion difficulty measures is still within the limits of the suitability of these measures. The interrater reliability results showed a substantial level of agreement between the raters at 55.6%, exceeding the 50% threshold. This underlines the success of the rater training sessions in establishing a common perspective for assessing assessment quality [27].

### 3.2 3CR's menus and criteria measurement (RQ2)

Table 4 shows the quality of logit measures for each assessed menu (*Content, Discussion Forum, Project Result, Test, Questionnaire*, and *Learning Reflection*), regarding an assessment rubric adjusted to the compiled criteria.

**Table 4:** Measurement result for the 3CR's menu facet.

Menu	Total Score	Total Count	Measure	Model S.E.	Infit		Outfit		Corr. PtMea
					MnSq	ZStd	MnSq	ZStd	
Discussion Forum (DF)	76	20	1.64	0.62	0.69	−0.60	0.62	−0.30	0.59
Project Result (PR)	88	25	0.72	0.44	1.08	0.30	1.04	0.20	0.56
Questionnaire (Q)	86	25	0.35	0.42	1.05	0.20	0.94	0.00	0.64
Content (C)	69	20	−0.22	0.45	0.60	−1.60	0.56	−1.50	0.55
Learning Reflection (LR)	66	20	−0.80	0.43	1.36	1.20	1.26	0.90	0.56
Test (T)	72	25	−1.69	0.37	1.01	0.10	1.02	0.10	0.69

**Fig. 1:**
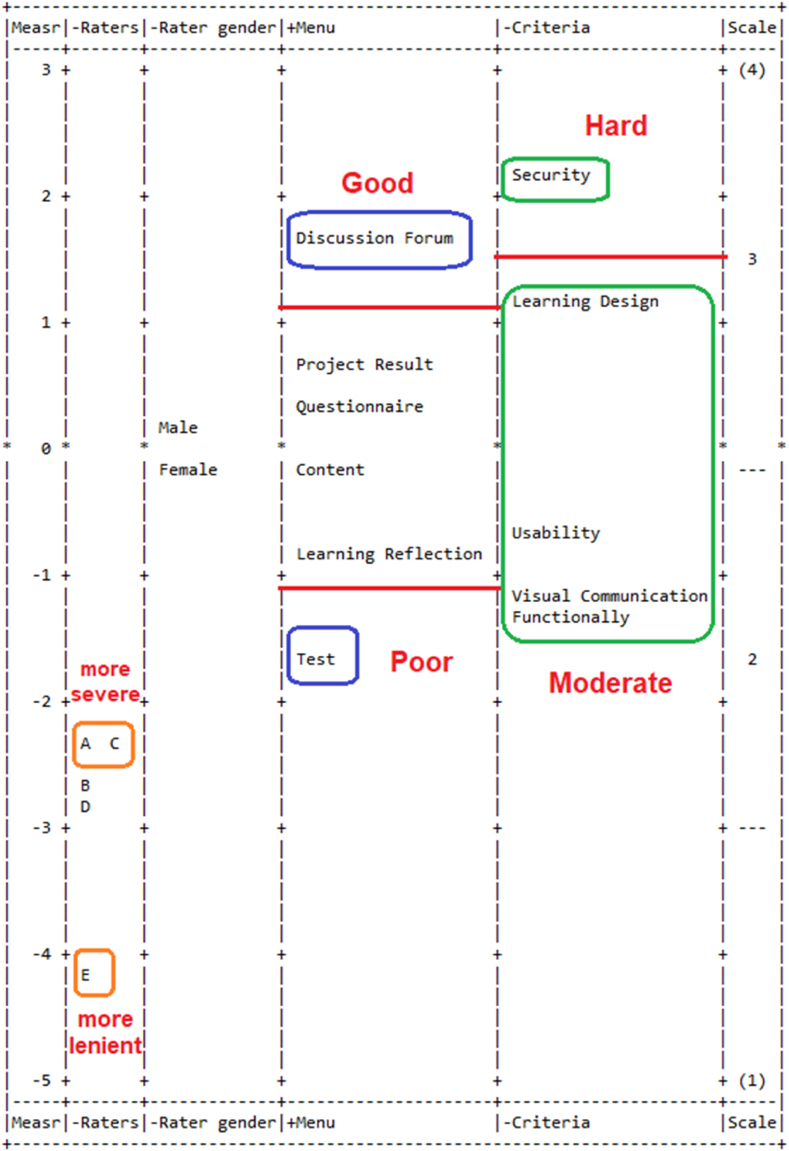
3CR menu quality, criteria, rater gender, and rater severity distribution.

Based on Table 4, the *Discussion Forum* (DF) had the highest logit value (+1.64;SE0.62), accompanied by the *Project Result* (PR), *Questionnaire* (Q), *Content* (C), *Learning Reflection* (LR), and *Test* (T) menus. According to the previous description, the logit value is a scale measurement unit used for each parameter specified in the Rasch model measurement. Fig. 1 also indicates a Wright Map graph, which presents the qualities (menu and criteria) of each 3CR application menu and the FACETS-based raters' severity and raters' gender. In this context, the first column represents the ruler scale on the left, ranging from −5 to +3. The scale units are in logits, with a reference point at zero. The second column indicates the distal facet, which refers to the gender of the raters. The third and fifth columns provided the distribution of each menu and criteria item, respectively. This explained how the distribution of the two columns was derived from the rater's response to the 3CR's menu regarding the analytical criteria's standard items.

Meanwhile, the fourth column indicates the range of rater severity or leniency. The measures associated with rater gender were close to zero, suggesting that rater gender may have little to no effect on rater ratings. Female raters exhibit a +0.24 logits, while male raters show −0.24 logits, confirming that male raters tend to be more stringent than female raters. The final column represents the scale criteria, encompassing a broad range from 1 to 4. Table 6 provides an explanation of how these scale criteria operate.

Based on Fig. 1 and Table 4, the 3CR's menu's distribution logit is within −1.69 to +1.64 and sorted from the lowest to the highest with mean values of 0.00 and SD 1.07 (see Table 3). The Discussion Forum menu has the highest logit (+1.64 logit), while the Test menu has the lowest one (−1.69 logit) (see the fourth column). The SD (Standard Deviation) value is essential to classify the quality of 3CR's menu. The 3CR's menu with logit higher than the SD demonstrates good quality of the menu, the 3CR's menu with logit lower than -SD has a poor quality of the menu, and those with the in-between demonstrate the moderate quality of the menu [27,50,52].

The fourth column in Fig. 1 shows the Discussion Forum menu with the best quality in the 3CR application according to the rater judgment. The other four 3CR's menus (Project Result, Questionnaire, Content, and Learning Reflection) have moderate quality, while the Test menu is poor quality. This categorization of 3CR's menu into three groups is supported by the index separation value (Table 3) for the menu.

The fifth column (“Criteria”) compares the five scoring criteria relative to their difficulties. Criteria listed higher in the column were found to be more difficult than those listed lower. In other words, when a particular criterion had a higher measure of difficulty, it was more difficult for menus to achieve a high score according to the raters' judgment [27,32]. *Security* was similarly high in difficulty, whereas other criteria (Learning Design, Usability, Visual Communication, and Functionality) were much less complex (moderate criterion). *Security* was the most challenging criterion item based on rater judgment (+1.63 logit). The high logit value on the *Security* criteria shows the result that the security system of the 3CR application needs to be considered again. Assessment related to the security system is not enough to do a trial use of the application. Raters need additional assessments to analyze whether it is true that the 3CR application supports user security. In light of this skepticism, the raters find it challenging to assess the security criteria optimally.

Meanwhile, “Functionality” and “Visual Communication” were the moderate items criteria approved by the raters at −1.21 logit and −1.39 logit, respectively. This was understandable because the appraisers were accustomed to using various tools to develop applications according to their respective fields of expertise, educational backgrounds, and more than ten years of work experience. Fig. 1 subsequently showed the rating severity levels (last column), where the most challenging and accessible ratings were exhibited at the top and bottom of the logit scale, respectively. The most difficult ratings are shown at the lowest scale category (below scale 1), and the most accessible rating is shown at the highest scale category (scale 4). Both scale categories represent extreme ratings (shown in parentheses only). This is because the thresholeds of the two extreme categories are (for the lowest one) and (for the highest one) [27].

In the analytical process, an accurate estimate of performance or quality was vital for valid measurement. This showed that MFRM analysis emphasized the provision of information about bias and unexpected responses from raters, regarding whether the ratings were very high (over-value) or low (under-value). Table 5 shows the differences in the leneincy or severity of the raters, which affects the estimation of the 3CR application menu quality obtained from the MFRM analysis FACETS program.

**Table 5:** Unexpected rater responses of 3CR menu rating.

Score	Expec	Rsd	StRes	Rater	Rater Gender	Menu	Criteria
2	3.9	−0.9	−3.5	B	Male	Discussion Forum	Visual Communication
2	3.7	−1.7	−3.4	B	Male	Questionnaire	Usability

Based on Table 5, only 2 of 135 ratings were provided by raters and were considered to contain some differential function. According to Table 5, the assessment score was “2". This was different from the expected (Expect) score of the model at 3.9, which was provided by rater B when rating the *“Discussion Forum”* menu for the *“Visual Communication”* criteria. Rater B has the expected (Expect) score of the mode at 3.7 when rating the *“Questionnaire”* menu for the *“Usability”* criteria. From these results, the assessment provided by rater B was distantly detected from the appropriate rating. The unexpected responses (Table 5) showed that the MRFM analysis predicted the consistency of each rater and the criterion item being scored for various menus. These results proved that total measurement capacity was carried out by MFRM analysis through the FACETS program.

Table 6 displays the statistics related to the rating categories, which describe the three-point scale ranging from “poor” to “excellent.” This substantiated the presence of a consistent enhancement concerning the average measures [53,54]. The Rasch-Andrich thresholds also showed a consistent improvement from low to 3.16 logits, indicating that the raters had a clear understanding of the rating scale categories [46]. For the Outfit MNSQ values, the poor, fair, good, and excellent category scales had 0.60, 0.90, 0.80, and 1.10 logits, respectively. This showed that the four categories had good fit validity [35,40]. Moreover, 1% (2), 8% (11), 43% (58), and 48% (65) were observed for Categories 1 (Strongly Insufficient), 2 (Insufficient), 3 (Sufficient), and 4 (Appropriate), respectively. This indicated the improvement in Categories 1 (Strongly Insufficient) to 4 (Appropriate), reflecting a high level of evaluating the menu quality of the 3CR application.

**Table 6:** Rating scale category statistics.

Rating scale category	Total	Percent	Average measure	Expected measure	Outfit MNSQ	Rasch-Andrich Thresholds
1(Strongly insufficient)	2	1%	−1.55	−1.02	0.60	low
2(Insufficient)	11	8%	0.43	0.34	1.00	−2.81
3(Sufficient)	58	43%	2.25	2.28	0.80	−0.35
4(Appropriate)	65	48%	4.22	4.22	1.10	3.16

### 3.3 Raters' severity and leniency (RQ3)

Severity estimates, their precision, and other relevant statistics are presented in Table 7.

**Table 7:** Measurement result for the rater facet.

Rater	Total	OA	FAM	M (logits)	SE	Infit MNSQ	Infit ZSTD	Outfit MNSQ	Infit ZSTD	PTMAC
C	90	3.33	3.23	−2.27	0.39	1.17	0.60	1.05	0.20	0.62
A	87	3.22	3.24	−2.33	0.37	0.65	−1.40	0.61	−1.50	0.76
B	89	3.30	3.32	−2.61	0.38	1.45	1.50	1.48	1.50	0.64
D	91	3.37	3.41	−2.91	0.39	0.82	−0.60	0.89	−0.20	0.68
E	100	3.70	3.72	−4.12	0.49	0.84	−0.30	0.55	−0.60	0.64

OA = Observed Average, FAM = Fair average measure, M = Measure, SE = Standard Error, PTMAC = Point measure correlation.

According to Table 7 and in the first column, raters appear in the orsder of their severity, that is, from most severe to most lenient. Rater A (FAM = 3.24, Measure = −2.33) was the most severe rater, while Rater E (FAM = 3.72, Measure = −4.12) was most lenient rater, respectively. According to their assessment, there were no significant differences between the actual average score and the FAM, indicating a high degree of accuracy in the raters' assessments. In addition, Fig. 1 (in the second column) shows a contrast between the raters in terms of the level of rigor each applied when rating the 3CRS menus. More severe raters appear higher in the column, while less severe (more lenient) raters appear lower; the rater facet has a negative orientation. Based on Fig. 1, raters with more severe evaluations are located at the top of the column (Rater A), and raters with more lenient evaluations are located at the bottom (Rater E). In Table 2, the rater strata and reliability were recorded as 2.09 and 0.63, respectively. Additionally, the chi-square statistic had a significant impact (χ^2^ = 123.7991; df = 5; *p* < .001). These findings indicated a significant level of diversity among raters, highlighting varying levels of stringency that influence the quality assessment of the 3CR application's menu.

Raters fit the Rasch model as determined by infit MNSQ, outfit MNSQ, and ZSTD. The expected values of the infit and outfit mean square statistics (MNSQ) are 1.0 and range from 0 to +∞. Raters with a fit value greater than 1.0 show more variation than expected in their ratings, called misfit (or underfit). By contrast, raters with fit values less than 1.0 show less variation than expected, indicating that their ratings are too predictable or provide redundant information; this is called overfitting [27]. Table 7 shows that the average infit and outfit MNSQ values for the rater facets are in the range of −4.12 logits to −2.27 logits, suggesting a problem of rater overfitting. Kim [55] and Jin & Eckes [56] stated that rater overfit often indicates the occurrence of central tendency or halo effects.

Further analysis is needed to ascertain whether the rater overfit is due to the central tendency effect or the halo effect. Analysis of the central tendency effect was conducted at the individual level. Rahman et al. (2017) stated that the central tendency effect at the individual level is more informative and valuable than at the group level. At the individual level, the central tendency effect can be detected using statistical categories for each rater: MNSQ Outfit and Threshold categories to generate hybrid model #1. This information can be obtained from Table 8. Outfit MNSQ greater than 1.5 indicates the presence of effects central tendency [57,58]. Outfit MNSQ for Rater B for the medium scale category is larger than the expected value, indicating more impact caused by the inspectors of central tendency than the other inspectors. Table 8 shows that the average threshold for Rater B is 1.97, which is ([1.79+2.15]/2). The mean threshold value for Rater B is the most significant compared to Raters A, C, D, and E (the mean threshold for them is 0.00). The extensive range reflects that the rater tends to use the scale more. The Outfit MNSQ analysis and average threshold analysis on Rater B show that Rater B tends to contribute to central tendency.

**Table 8:** Category statistic for rater B.

Score	Category Scale (%)	Outfit MNSQ	Threshold Measure
1	4	0.30	None
2	11	2.30	−1.79
3	37	1.30	−0.36
4	48	1.30	2.15

**Fig. 2:**
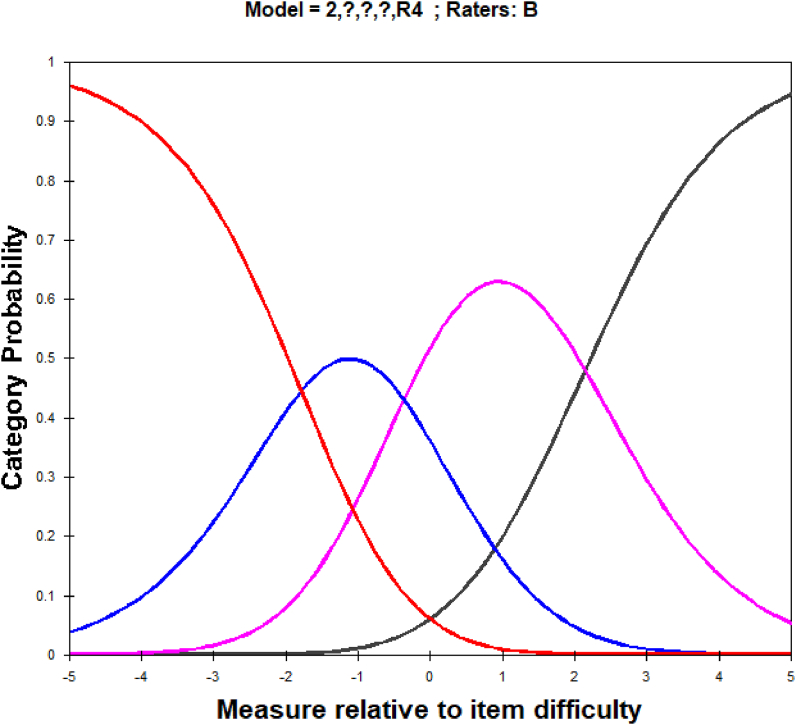
Category probability curves for centeral tendency Rater.

In the context of MFRM analysis, the randomness effect is defined as the tendency of a rater to apply one or more trait scales in a way that is inconsistent with the way other raters apply the same scales. A rater who experiences randomness needs to be more consistent in her use of the scales, showing more random variability than expected in her ratings (Rater B is Female), even after the quality of the 3CR menus the raters evaluated has been taken into account. Fig. 2 shows category probability curves for central tendency rater B.

### 3.4 Raters' gender bias in menu assessment (RQ4)

At the group level, three statistical indicators may provide information on gender bias [59,60]: (a) the fixed chi-square statistic to find out whether female and male raters shared the same calibrated level of performance, (b) the gender separation index, to determine the number of statistically distinct levels of performance among the gender groups, and (c) the reliability of gender separation, to see how well female and male rater were separated in terms of their performances.

As shown in Table 8, there were notable differences in the measures for the discussion forum menu between female raters (2.64 logits; SE = 1.54) and male raters (1.10 logits; SE = 0.66). As a result, there was a significant difference in logits (DIF size) with a substantial probability (p < .01). Table 8 further shows that the Learning Reflection Menu had a significant size difference between female and male raters (−1.61 logits; SE = 0.71), resulting in a goal contrast (DIF Size) of 1.29 logits with an elevated probability (p < .01). Similarly, the gender separation index was 1.79. The reliability of gender separation was 0.54.

Male raters are more severe than female raters in the Discussion Forum Menu and Learning Reflection Menu rating. The adjusted chi-square test examining the interaction of bias between menu and raters' gender is statistically significant (χ^2^(12) = 4.1; *p* < .01). This implies that the observed variation in measurement is substantial and not merely a chance occurrence. This result is critical, even though the menu and raters' gender interaction appears minor. However, in the presence of a significant bias within a particular rater's measurement, it becomes imperative to invalidate the final results to ensure fairness in the evaluation of 3CR's menu quality.

**Fig. 3:**
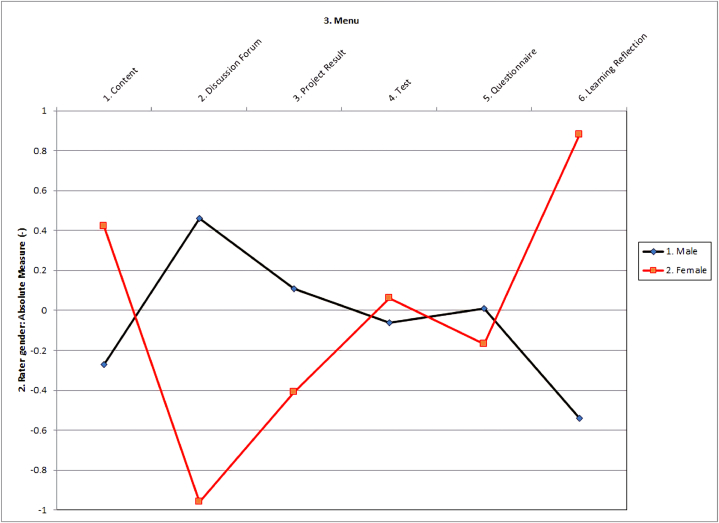
The DIF graph shows the interaction of rater gender bias and the 3CR menu.

We also used DIF analysis to validate any potential rater bias associated with the gender of the raters in the assessment. DIF analysis reveals subgroup differences in rater responses, particularly rater gender, for each of the 3CR menus within the rating [27,61]. The DIF graph illustrates the menu bias based on raters' gender in absolute terms. Conversely, the Discussion Forum Menu displayed notable variations in menu ratings based on rater's gender. Fig. 3 provides a visual representation of the interaction between raters' gender and menu bias.

Fig. 3 depicts the gender bias diagram for the entire set of 3CR menus. Gender bias among raters varies significantly across 3CR's menus. Male raters, for example, scored 3CR's menu lower than expected, whereas female raters did the opposite (see also Table 9).

**Table 9:** Bias interaction between raters' gender and 3CR's menu.

3CR's menu	Target measure (logits)	Raters' gender	Target measure (logits)	Raters' gender	Target contrast	t	d.f.	p
Content	−0.01	Male	−0.58	Female	0.57	0.61	14	0.5535
Discussion Forum	1.10	Male	2.64	Female	−1.55	−0.92	9	0.3810
Project Result	0.57	Male	1.22	Female	−0.65	−0.67	16	0.5135
Test	−1.75	Male	−1.74	Female	−0.01	−0.01	17	0.9887
Questionnaire	0.31	Male	0.62	Female	−0.31	−0.34	16	0.7391
Learning Reflection	−0.32	Male	−1.61	Female	1.29	1.44	14	0.1720

p = probability(significant if p < .01), t = bias-statistic, d.f = degree of freedom.

## 4 Discussion

Based on the results, all menu components in the 3CR application were valid and reliable for use in the learning process, using the Rasch parameters. This showed that raters had varying degrees of severity when assessing the learning application qualities. This information was corroborated by Meier et al. (2007), where raters had different size estimates in assessing the quality of an application. It also indicated that the assessment of process quality required a specific amount of raters' interpretation, leading to low objectivity when they are not carefully trained. Furthermore, Leacock & Nesbit [62] suggested that raters should consider an unusable object when rating the content quality of an application, regarding serious inaccuracies, biases, or omissions to warrant a ranking of 1.

According to the evaluation of menus in the assessment of the 3CR application, several results were obtained using MFRM analysis. The result showed that the Test menu had the lowest rating (Measure = −1.69), with the unexpected outputs of the rater's assessment subsequently observed; meanwhile, the Discussion Forum menu has the highest quality in the 3CR application. Good ratings from the rater on the Discussion Forum menu align with the objectives of the 3CR application's development, which provided flexibility in the implementation of collaborative, interactive, and tiered discussion activities according to the competencies and needs possessed by students. This result was corroborated by Tiria et al. [63] where Discussion Forum on online learning applications provided opportunities for students to conduct digital interactions. The existence of this flexible item further helps to increase students' active participation in discussion activities and is key to the success of learning implementation using digital application media. Wang [64] also stated that online discussion is an essential component of the digital learning environment and an important dimension of the learning process. Additionally, the unique capabilities of online discussion exhibited a platform to support student learning and broadly promoted collaborative and meaningful academic processes.

In the application menu, especially Discussion Forum, the support for the ethno-flipped classroom model provided a different quality effect compared to similar learning tools. This support subsequently provided better quality tools for other menus in the 3CR application. These results were observed in the unexpected assessment provided by raters using the FACETS program having only 2 of 135 ratings. Based on the result, the analysis showed that the support between the menus contained in the 3CR application was excellent, indicating that the rater had no difficulty providing a rating on the established assessment criteria.

The criteria measurement results in Table 2 provide results where three criteria items observed on the menu were not included in the data analysis, so the number of data analyzed was 135 data points. However, the overall menu measurement analysis shows that the quality of the menu developed in this application is valid and reliable (fulfills the fit validity analysis). Furthermore, the developed 3CT application also shows its appropriate utilization in learning, especially in applying the ethno-flipped classroom model.

This analysis further confirmed that Security was the most challenging item for the raters to assess. The rater's difficulty in assessing the Security item is because the rater still needs additional information related to the cloud system used and the user's security procedures in accessing the application. Security in an application is related to the security of the data stored in the application and the information obtained by users when accessing the application. This result shows that Security must be considered before developing an application [65,66].

The raters also provided suggestions and input related to the assessment of the Security criteria, including:

1) The certainty that the application can be accessed from anywhere must also be a concern to fulfill the application's availability. By connecting the system to computer networks and the internet, the opportunity to change or damage data will be more widely open because users of 3CR application that are potentially dangerous (malicious users) will quickly enter the system through computer networks/internet.
2) The researchers necessary to apply content protection to e-learning, which refers to protecting the integrity and copyright of the subject matter. Meanwhile, the protection of web-based applications is needed because attacks have increased rapidly in recent years.

The MFRM analysis answered another research question relating to the severity/leniency of the raters in rating the 3CR menu. The analysis showed that Rater A was more severe and Rater E was more lenient in rating the 3CR menu. However, the results of the analysis also show the presence of rater overfitting, which appears in Rater B and indicates the occurrence of central tendency effect. Several studies found that rater effects impact on the measurement quality of assessment outcomes [[67], [68], [69]].

Identifying the source of bias in evaluation results is a complex task. Raters must strive for greater consistency in evaluating both theoretical concepts and presenter backgrounds [55,61]. This study demonstrated that MFRM analysis can assist researchers in detecting bias interaction related to rater gender when evaluating the 3CR's menu. The results show that Male raters are more severe than female raters in the Discussion Forum Menu and Learning Reflection Menu rating.

## 5 Study limitations and recommendation for future analysis

This research has limitations in several ways. The first is the limited amount of data to analyze. We formulated the combination dataset using five raters, six menus, and five criteria only, so this study has limitations on the analysis data, which is 150 data points. Nevertheless, we encourage other researchers to undertake assessments of the quality of the developed application menus and include additional raters with a similar gender distribution. Increasing the number of raters will help researchers obtain more varied quality data and minimize the chance of rater gender bias on the menu being assessed. Second, adding other distal facets such as educational background, experience developing similar applications, and other facets related to the rater in the analysis process will provide more detailed analysis results to show that the developed menus have good quality. Third, the determination of assessment criteria in this study still uses only five criteria, so we recommend other researchers use more criteria so that the assessment of application menu quality can provide better results. Many-Facet Rasch Model analysis has been widely applied in assessing a person's performance through expert judgment. However, research that analyzes application quality assessment through expert assessment using the Many-Facet Rasch Model analysis are rarely used in educational contexts. Based on this, we hope that this research will serve as a catalyst to encourage other researchers to undertake similar studies, thereby contributing to the expansion of knowledge about the evaluation of the quality of learning applications.

## 6 Conclusion

The Collaborative Cloud Classroom (3CR) application was used to help the learning process, with the quality of its good menu measured by five test criteria items. These items were derived from the analysis of the standards utilized by the raters acting as study raters. This was conducted using the Many-Facet Rasch Model analysis, which simultaneously measured the qualities of the application menu and criteria items, as well as the level of acuity or severity of expert judgment. Based on the results, the 3CR application menu had good quality, which was obtained from the unit logit value, using the FACETS program. This was observed from the distribution of the application menu quality, where the *Discussion Forum* (DF) had the best value. Regarding the five items of test criteria, “*Security*” was the most difficult criterion for the appraisers to agree on.

Moreover, the study found that Rater A was more severe in evaluating the instrument than other raters, and Rater E was more lenient in evaluating the instrument than other raters in rating the 3CR menu. There was rater overfitting (Rater B) and detected bias between rater gender and 3CR's menu (Discussion Forum and Learning Reflection menu) based on MFRM analysis. This study demonstrated that MFRM analysis could effectively assess the quality of 3CR's menu and the severity/leniency rater, rater bias, and bias interaction in rater gender in assessing learning application.

## Data availability statement

Data will be made available on request.

## CRediT authorship contribution statement

**Rahmi Ramadhani:** Conceptualization, Formal analysis, Investigation, Methodology, Software, Validation, Visualization, Writing – original draft, Writing – review & editing. **Edi Syahputra:** Conceptualization, Methodology, Resources, Supervision, Validation, Writing – review & editing. **Elmanani Simamora:** Conceptualization, Data curation, Investigation, Supervision, Validation, Writing – review & editing. **Soeharto Soeharto:** Data curation, Formal analysis, Investigation, Methodology, Resources, Software, Visualization, Writing – review & editing.

## Declaration of competing interest

The authors declare that they have no known competing financial interests or personal relationships that could have appeared to influence the work reported in this paper.
