# Supplementary material for: Expert judgement of collaborative cloud classroom quality and its criteria using the many-facets rasch model
Source: Heliyon. 2023 Oct 5;9(10):e20596. doi: 10.1016/j.heliyon.2023.e20596 (PMC10570598; doi:10.1016/j.heliyon.2023.e20596)
Supplement: Multimedia component 1 [file mmc1.docx]

**Appendix-A**

**Collaborative Cloud Classroom Application Quality Assessment Instrument**

| **Menu** | **Criteria** | **Scoring Rubric** | | | | **Score** |
| --- | --- | --- | --- | --- | --- | --- |
|  |  | **Collaborative Cloud Classroom Application** | | | |  |
| Content (C) | Usability (U) | **1** | **2** | **3** | **4** |  |
|  |  | The content menu cannot be accessed easily and cannot display the menu page quickly. | The content menu cannot be accessed easily and cannot display the menu page quickly. | The content menu can be accessed easily, but it lacks the ability to display the menu page quickly. | The content menu can be accessed easily and can display the menu page quickly. |  |
|  | Functionality (F) | **1** | **2** | **3** | **4** |  |
|  |  | The content menu in the Collaborative Cloud ClassRoom application cannot function properly and optimally. | The content menu in the Collaborative Cloud ClassRoom application can function less well and optimally. | The content menu in the Collaborative Cloud ClassRoom application can function properly and optimally. | The content menu in the Collaborative Cloud ClassRoom application can function properly and optimally. |  |
|  | Visual Communication (VC) | **1** | **2** | **3** | **4** |  |
|  |  | The content menu uses a poorly structured display design and uncommunicative language. | The content menu uses a less well-structured display design and less communicative language. | The content menu uses a well-structured display design, but the language is less communicative. | The content menu uses a well-structured display design and communicative language. |  |
|  | Learning Design (LD) | **1** | **2** | **3** | **4** |  |
|  |  | Learning design on the content menu does not support the implementation of ethno-flipped classroom model syntax. | The learning design on the content menu does not support the implementation of the syntax of the ethno-flipped classroom model. | The learning design on the content menu supports the implementation of the syntax of the ethno-flipped classroom model, but not yet optimal. | The learning design on the content menu supports the implementation of the syntax of the ethno-flipped classroom model optimally. |  |
|  | Security (S) | **1** | **2** | **3** | **4** |  |
|  |  | The developed content menu does not have a secure registration menu. | The developed content menu has a registration menu that is less secure and less easy to use. | The developed content menu has a secure registration menu, but is less easy to use. | The developed content menu has a secure and easy-to-use registration menu. |  |
| Discussion Forum (DF) | Usability (U) | **1** | **2** | **3** | **4** |  |
|  |  | The Discussion Forum menu cannot be accessed easily and cannot display the menu page quickly. | Discussion Forum menu is less accessible and less able to display the menu page quickly. | The Discussion Forum menu can be accessed easily, but is less able to display the menu page quickly. | Discussion Forum menu can be accessed easily and can display the menu page quickly. |  |
|  | Functionality (F) | **1** | **2** | **3** | **4** |  |
|  |  | The Discussion Forum menu in the Collaborative Cloud ClassRoom application cannot function properly and optimally. | The Discussion Forum menu in the Collaborative Cloud ClassRoom application can function less well and optimally. | The Discussion Forum menu in the Collaborative Cloud ClassRoom application can function properly and optimally. | The Discussion Forum menu in the Collaborative Cloud ClassRoom application can function properly and optimally. |  |
|  | Visual Communication (VC) | **1** | **2** | **3** | **4** |  |
|  |  | The Discussion Forum menu uses a display design that is not well structured and uncommunicative language. | The Discussion Forum menu uses a display design that is less well structured and less communicative language. | The Discussion Forum menu uses a well-structured display design, but the language is less communicative. | The Discussion Forum menu uses a well-structured display design and communicative language. |  |
|  | Learning Design (LD) | **1** | **2** | **3** | **4** |  |
|  |  | Learning design on the Discussion Forum menu does not support the implementation of ethno-flipped classroom model syntax. | The learning design on the Discussion Forum menu does not support the implementation of the syntax of the ethno-flipped classroom model. | Learning design on the Discussion Forum menu supports the implementation of ethno-flipped classroom model syntax, but not yet optimal. | The learning design on the Discussion Forum menu supports the implementation of the syntax of the ethno-flipped classroom model optimally. |  |
|  | Security (S) | **1** | **2** | **3** | **4** |  |
|  |  | The developed Discussion Forum menu does not have a secure registration menu. | The developed Discussion Forum menu has a registration menu that is less secure and less easy to use. | The developed Discussion Forum menu has a secure registration menu, but is less easy to use. | The developed Discussion Forum menu has a secure and easy-to-use registration menu. |  |
| Project Result (PR) | Usability (U) | **1** | **2** | **3** | **4** |  |
|  |  | The Project Result menu cannot be accessed easily and cannot display the menu page quickly. | The Project Result menu is less accessible and less able to display menu pages quickly. | The Project Result menu can be accessed easily, but is less able to display the menu page quickly. | The Project Result menu can be accessed easily and can display the menu page quickly. |  |
|  | Functionality (F) | **1** | **2** | **3** | **4** |  |
|  |  | The Project Result menu in the Collaborative Cloud ClassRoom application cannot function properly and optimally. | The Project Result menu in the Collaborative Cloud ClassRoom application can function less well and optimally. | The Project Result menu in the Collaborative Cloud ClassRoom application can function properly and optimally. | The Project Result menu in the Collaborative Cloud ClassRoom application can function properly and optimally. |  |
|  | Visual Communication (VC) | **1** | **2** | **3** | **4** |  |
|  |  | The Project Result menu uses a display design that is not well structured and uncommunicative language. | The Project Result menu uses a less well-structured display design and less communicative language. | The Project Result menu uses a well-structured display design, but the language is less communicative. | The Project Result menu uses a well-structured display design and communicative language. |  |
|  | Learning Design (LD) | **1** | **2** | **3** | **4** |  |
|  |  | The learning design on the Project Result menu does not support the implementation of the syntax of the ethno-flipped classroom model. | The learning design on the Project Result menu does not support the implementation of the syntax of the ethno-flipped classroom model. | The learning design on the Project Result menu supports the application of ethno-flipped classroom model syntax, but not yet optimal. | The learning design on the Project Result menu supports the syntax of the ethno-flipped classroom model optimally. |  |
|  | Security (S) | **1** | **2** | **3** | **4** |  |
|  |  | The developed Project Result menu does not have a secure registration menu | The developed Project Result menu has a registration menu that is less secure and less easy to use. | The developed Project Result menu has a secure registration menu, but is less easy to use. | The developed Project Result menu has a secure registration menu and is easy to use. |  |
| Test (T) | Usability (U) | **1** | **2** | **3** | **4** |  |
|  |  | The Test menu cannot be accessed easily and cannot display the menu page quickly. | The Test menu is less accessible and less able to display menu pages quickly. | The Test menu can be accessed easily, but is less able to display menu pages quickly. | The Test menu can be accessed easily and can display the menu page quickly. |  |
|  | Functionality (F) | **1** | **2** | **3** | **4** |  |
|  |  | The Test menu in the Collaborative Cloud ClassRoom application cannot function properly and optimally. | The Test menu in the Collaborative Cloud ClassRoom application can function less well and optimally. | The Test menu in the Collaborative Cloud ClassRoom application can function properly and optimally. | The Test menu in the Collaborative Cloud ClassRoom application can function properly and optimally. |  |
|  | Visual Communication (VC) | **1** | **2** | **3** | **4** |  |
|  |  | The Test menu uses a display design that is not well structured and uncommunicative language. | The Test menu uses a display design that is less well structured and less communicative language. | The Test menu uses a well-structured display design, but the language is less communicative. | The Test menu uses a well-structured display design and communicative language. |  |
|  | Learning Design (LD) | **1** | **2** | **3** | **4** |  |
|  |  | The learning design on the Test menu does not support the implementation of the syntax of the ethno-flipped classroom model. | The learning design on the Test menu does not support the implementation of the syntax of the ethno-flipped classroom model. | The learning design on the Test menu supports the implementation of the syntax of the ethno-flipped classroom model, but not yet optimal. | The learning design on the Test menu supports the implementation of the syntax of the ethno-flipped classroom model optimally. |  |
|  | Security (S) | **1** | **2** | **3** | **4** |  |
|  |  | The developed Test menu does not have a secure registration menu. | The developed Test menu has a registration menu that is less secure and less easy to use. | The developed Test menu has a secure registration menu, but is less easy to use. | The developed Test menu has a secure and easy-to-use registration menu. |  |
| Questionnaire (Q) | Usability (U) | **1** | **2** | **3** | **4** |  |
|  |  | Questionnaire menu cannot be accessed easily and cannot display the menu page quickly. | Questionnaire menu is less accessible and less able to display menu pages quickly. | Questionnaire menu is less accessible and less able to display menu pages quickly. | Questionnaire menu can be accessed easily and can display the menu page quickly. |  |
|  | Functionality (F) | **1** | **2** | **3** | **4** |  |
|  |  | The Questionnaire menu in the Collaborative Cloud ClassRoom application cannot function properly and optimally. | The Questionnaire menu in the Collaborative Cloud ClassRoom application can function less well and optimally. | The Questionnaire menu in the Collaborative Cloud ClassRoom application can function properly and optimally. | The Questionnaire menu in the Collaborative Cloud ClassRoom application can function properly and optimally. |  |
|  | Visual Communication (VC) | **1** | **2** | **3** | **4** |  |
|  |  | The Questionnaire menu uses a display design that is not well structured and uncommunicative language. | Questionnaire menu uses a display design that is less well structured and less communicative language. | Questionnaire menu uses a well-structured display design, but the language is less communicative. | Questionnaire menu uses a well-structured display design and communicative language. |  |
|  | Learning Design (LD) | **1** | **2** | **3** | **4** |  |
|  |  | The learning design on the Questionnaire menu does not support the implementation of the syntax of the ethno-flipped classroom model. | The learning design on the Questionnaire menu does not support the implementation of the syntax of the ethno-flipped classroom model. | The learning design on the Questionnaire menu supports the implementation of the syntax of the ethno-flipped classroom model, but not yet optimal. | The learning design on the Questionnaire menu supports the implementation of the syntax of the ethno-flipped classroom model optimally. |  |
|  | Security (S) | **1** | **2** | **3** | **4** |  |
|  |  | The developed Questionnaire menu does not have a secure registration menu. | The developed Questionnaire menu has a registration menu that is less secure and less easy to use. | The developed Questionnaire menu has a secure registration menu, but is less easy to use. | The developed Questionnaire menu has a secure registration menu and is easy to use. |  |
| Learning Reflection (LR) | Usability (U) | **1** | **2** | **3** | **4** |  |
|  |  | The Learning Reflection menu cannot be accessed easily and cannot display the menu page quickly. | The Learning Reflection menu is less accessible and less able to display menu pages quickly. | The Learning Reflection menu can be accessed easily, but is less able to display menu pages quickly. | The Learning Reflection menu can be accessed easily and can display the menu page quickly. |  |
|  | Functionality (F) | **1** | **2** | **3** | **4** |  |
|  |  | The Learning Reflection menu in the Collaborative Cloud ClassRoom application cannot function properly and optimally. | The Learning Reflection menu in the Collaborative Cloud ClassRoom application can function less well and optimally. | The Learning Reflection menu in the Collaborative Cloud ClassRoom application can function properly and optimally. | The Learning Reflection menu in the Collaborative Cloud ClassRoom application can function properly and optimally. |  |
|  | Visual Communication (VC) | **1** | **2** | **3** | **4** |  |
|  |  | The Learning Reflection menu uses a poorly structured display design and uncommunicative language. | The Learning Reflection menu uses a less well-structured display design and less communicative language. | The Learning Reflection menu uses a well-structured display design, but the language is less communicative. | The Learning Reflection menu uses a well-structured display design and communicative language. |  |
|  | Learning Design (LD) | **1** | **2** | **3** | **4** |  |
|  |  | Learning design on the Learning Reflection menu does not support the implementation of ethno-flipped classroom model syntax. | The learning design on the Learning Reflection menu does not support the implementation of the syntax of the ethno-flipped classroom model. | The learning design on the Learning Reflection menu supports the implementation of the syntax of the ethno-flipped classroom model, but not yet optimal. | The learning design on the Learning Reflection menu supports the implementation of the ethno-flipped classroom model syntax optimally. |  |
|  | Security (S) | **1** | **2** | **3** | **4** |  |
|  |  | The developed Learning Reflection menu does not have a secure registration menu. | The developed Learning Reflection menu has a registration menu that is less secure and less easy to use. | The developed Learning Reflection menu has a secure registration menu, but is less easy to use. | The developed Learning Reflection menu has a secure and easy-to-use registration menu. |  |
