# Supplementary material for: Expert judgement of collaborative cloud classroom quality and its criteria using the many-facets rasch model
Source: Heliyon. 2023 Oct 5;9(10):e20596. doi: 10.1016/j.heliyon.2023.e20596 (PMC10570598; doi:10.1016/j.heliyon.2023.e20596)
Supplement: Multimedia component 2 [file mmc2.docx]

**Appendix-B**

TITLE = "Validity of 3CR Application"

Facets = 4; Raters + raters gender + Menu + Criteria

Inter-rater = 1; computer inter-rater agreements (inter-rater = rater facet number)

Positive = 1, 3; the first and third facet has positive ability

Non-centered = 1; measure from the center of the measure for each facet

Left=Yes

Model =

#,?,?,?,R4 ; put the model statement for your facets and elements here

?B,?,?B,?,R4 ; bias interaction between raters and menu

?,?B,?B,?,R4 ; bias interaction between raters gender and Menu

?,?B,?,?B,R4 ; bias interaction between raters gender and Criteria

*

Labels =

*

1, Raters

1 = A

2 = B

3 = C

4 = D

5 = E

*

2, Rater gender, A

1 = Male

2 = Female

*

3, Menu, A

1 = Content

2 = Discussion Forum

3 = Project Result

4 = Test

5 = Questionnaire

6 = Learning Reflection

*

4, Criteria

1 = Usability

2 = Functionally

3 = Visual Communication

4 = Learning Design

5 = Security

*

Data=

1,1,1,1-5,3,3,4,3,

1,1,2,1-5,4,4,4,3,

1,1,3,1-5,3,4,4,3,3

1,1,4,1-5,4,3,3,3,2

1,1,5,1-5,3,4,4,3,2

1,1,6,1-5,3,3,3,2,

2,1,1,1-5,3,4,4,3,

2,1,2,1-5,4,4,3,3,

2,1,3,1-5,4,4,4,3,2

2,1,4,1-5,4,3,3,2,1

2,1,5,1-5,2,4,4,3,3

2,1,6,1-5,3,4,4,4,

….

5,2,1,1-5,4,4,4,3,

5,2,2,1-5,4,4,4,4,

5,2,3,1-5,4,4,4,3,3

5,2,4,1-5,3,3,3,3,3

5,2,5,1-5,4,4,4,4,4

5,2,6,1-5,4,4,4,4,
